# Moonlight Makes Owls More Chatty

**DOI:** 10.1371/journal.pone.0008696

**Published:** 2010-01-20

**Authors:** Vincenzo Penteriani, María del Mar Delgado, Letizia Campioni, Rui Lourenço

**Affiliations:** 1 Department of Conservation Biology, Estación Biológica de Doñana, C.S.I.C., Seville, Spain; 2 Finnish Museum of Natural History, Zoological Museum, University of Helsinki, Helsinki, Finland; 3 Laboratory of Ecological and Evolutionary Dynamics, Department of Biological and Environmental Sciences, University of Helsinki, Helsinki, Finland; 4 Institute of Mediterranean Agricultural Sciences (ICAM), University of Évora, Évora, Portugal; University of Hull, United Kingdom

## Abstract

**Background:**

Lunar cycles seem to affect many of the rhythms, temporal patterns and behaviors of living things on Earth. Ambient light is known to affect visual communication in animals, with the conspicuousness of visual signals being largely determined by the light available for reflection by the sender. Although most previous studies in this context have focused on diurnal light, moonlight should not be neglected from the perspective of visual communication among nocturnal species. We recently discovered that eagle owls *Bubo bubo* communicate with conspecifics using a patch of white throat plumage that is repeatedly exposed during each call and is only visible during vocal displays.

**Methodology/Principal Findings:**

Here we provide evidence that this species uses moonlight to increase the conspicuousness of this visual signal during call displays. We found that call displays are directly influenced by the amount of moonlight, with silent nights being more frequent during periods with no-moonlight than moonlight. Furthermore, high numbers of calling bouts were more frequent at moonlight. Finally, call posts were located on higher positions on moonlit nights.

**Conclusions/Significance:**

Our results support the idea that moon phase affects the visual signaling behavior of this species, and provide a starting point for examination of this method of communication by nocturnal species.

## Introduction

Lunar cycles appear to regulate many of the cycles and temporal patterns that govern life on Earth, such as the migratory, reproductive and hunting behavior of many species [1]–[8]. Moreover, because bright moonlight may increase the risk of predation by visually-oriented predators, lunar-related activity patterns have been described for a number of nocturnal mammals and birds (i.e. lunar phobia [9]–[13]).

Visual signals are more or less conspicuous depending on the amount of light available for reflection [14], [15], but the numerous studies that have assessed the relationships between feather coloration and light environment [14] have considered only diurnal light. However, moonlight represents a powerful source of illumination that cannot be neglected from the perspective of visual communication in nocturnal species [16]. The luminance of a full moon (∼0.25 lux) is approximately 25 times greater than that of the quarter moon and 250 times greater than that of a moonless clear starry night sky [17]. As recently pointed out by Théry [14] “…*light environments are just beginning to garner attention*, *and several questions are not answered*, *if even asked*.” One such question is: does moonlight affect animal communication?

Owl visual sensitivity permits some degree of vision under naturally-occurring nocturnal conditions, but nocturnal vision is best under moonlight [18]. Eagle owls *Bubo bubo* use visual signaling in intraspecific communication: their white throat badge is repeatedly exposed at each call and it is only visible during vocal displays [19]–[21]. Because of the important role played by the visual communication in this nocturnal species, we suggest that white plumage patches (achromatic plumage patches are the best candidates for night-time signaling, when contrast is more important than color) and the timing of visual signaling may have co-evolved to maximize effectiveness of the signal. Under such a scenario, we can expect that nocturnal birds with conspicuous white feathers: (a) will call more at full moon, when the lunar light favors communication via visual signaling (visual displays and vocal bouts represent multimodal signaling); and (b) if lunar brightness facilitates owl visual communication, displaying individuals should select higher call posts at full moon, because higher positions increase the conspicuousness of signal.

## Methods

From 2003 to 2008, we radiotagged 26 breeding eagle owls (n = 14 males; n = 9 females) from 16 breeding sites in south-western Spain [20]. Each individual was fitted with a 30-g radio-transmitter using a Teflon ribbon backpack harness. Transmitters had a mercury posture sensor that allowed us to discriminate activity changes in the radiosignal. The mass of the backpack corresponded to less than 3% of the mass of the smallest adult male (1,550 g) in our population (mean ± SE: 1,667±104.8 g). We trapped breeding males by simulating a territorial intrusion using a taxidermic mount and a playback of a male call. A heavy duty mist-net behind the mount caught responding individuals. To avoid female disturbance during incubation and the nestling stage (when male trapping occurred), mounts were always positioned >100 to the nest and in a position not visible to the female. Females (as well as some males) were trapped with a bownet placed in the nest when nestlings were from 20 to 35 days-old, i.e. when they could thermoregulate and night temperatures were warm (about 20°C). Nestlings were put in a box with a metal grid making them visible to their parents, who were caught when they returned to the nest. After each bownet trapping session (which lasted from sunset to sunrise), we fed nestlings and released them in the nest. We never performed more than three trapping nights per breeding season in the same nest. After 7 years of trapping and continuous radiotracking of more than 150 individuals (both breeders and floaters), we never recorded adverse effects due to captures and backpacks on birds, reproduction or site fidelity (Penteriani & Delgado, unpublished data). Backpacks were not removed due to the difficulty of recapturing the same individual (Penteriani & Delgado unpublished data). Owls were sexed using DNA extracted from blood. All research involving animals has been conducted according to relevant national and international guidelines: we manipulated and marked owls under Junta de Andalucía – Consejería de Medio Ambiente permits No. SCFFS-AFR/GGG RS-260/02 and SCFFS-AFR/CMM RS-1904/02.

### Lunar Phases

**Figure 1:**
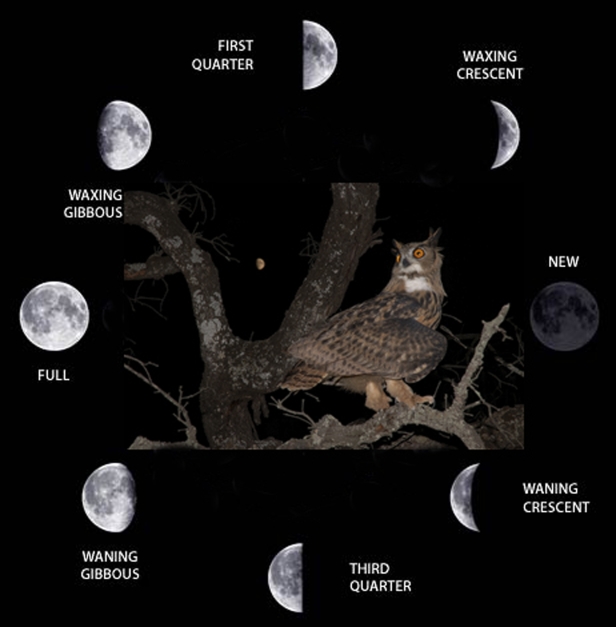
The five lunar phases that have been considered in the analyses (see the text for more details): new moon, waxing/waning crescent, first/third quarter, waxing/waning gibbous and full moon. The waxing/waning phases and first/third quarter moons (often called a “half moon”) have been grouped together because of their equivalent illumination (http://aa.usno.navy.mil/faq/docs/moon_phases).

Considering the moon as a circular disk, the ratio of the area illuminated by direct sunlight to its total area is the fraction of the moon's surface illuminated, which multiplied by 100 represent the percent illuminated (http://aa.usno.navy.mil/faq/docs/moon_phases). Consequently, moons were grouped into five lunar phases on the basis of the percent illuminated: (phase 0) new moon, the percent illuminated is 0–10%; (phase 1) waxing/waning crescent, the percent illuminated is between 11 and 25%; (phase 2) first/third quarter, the percent illuminated is between 26 and 50%; (phase 3) waxing/waning gibbous (the percent illuminated is between 51% and 90%); and (4) full moon (the percent illuminated is between 91 and 100%). The waxing/waning phases and first/third quarter moons have been grouped together because of their equivalent illumination (Fig. 1). The first (waxing moon) and the third (waning moon) quarters, plus the night of the full moon itself, comprise the brightest nights of each month.

As a general rule, we never performed a radiotracking night under adverse weather conditions. However, and specifically for the scope of this work, our analyses excluded windy and rainy nights, as well as nights during which the moon was hidden by clouds, to avoid potential biases due to (a) interferences of bad weather conditions on behavior and (b) different levels of ambient light at night [18], [8].

### Individual Tracking

We performed 189 nights of continuous radiotracking (66 moonlight and 123 no-moonlight), uniformly distributed among individuals and across the year (2040.3 hrs of radiotracking). A continuous radiotracking session was defined as following a focal individual from 1 hr before sunset to 1 hr after sunrise; the mean duration of radiotracking sessions = 11.3±2.1 hrs. During continuous radiotracking sessions, we recorded a location each time we detected a change in the signal, indicating a change in the individual's posture and/or position. Signals were detected by a fixed antenna on the roof of a car. Locations were triangulated with a 3-element hand-held Yagi-antenna connected to portable receivers from ICOM (IC-R20).

### Data Analyses

We used Generalized Linear Mixed Models to test for moonlight effects on (a) call activity (i.e. whether or not owls called during the night); (b) number of call bouts (i.e. series of single *oohu*) per night. The explanatory variables included: (1) moon phases (the five classes described above); (2) year; (3) sex; and (4) breeding cycle, i.e. pre-laying (the whole of the period between dispersal one year and laying the next year), incubation, nestling, and fledgling to natal dispersal periods [22]. Individuals were considered as a random effect in Generalized Linear Mixed Models, because we had repeated-measures of the same individuals, and to avoid pseudoreplication. The response variables did not have a normal distribution; therefore call activity and number of call bouts (Poisson distributions) were modeled using log link functions. The statistical analyses were performed using GLIMMIX (SAS), which iterates the procedure, MIXED. To test whether call posts were selected as a function of moonlight, we used Arc View v 3.2 geographic information system (GIS) software to obtain map representations (1∶25 000) of the 306 different call posts recorded during radiotracking, and compared the coefficient of dominance [23] between posts selected during moonlight *vs*. no-moonlight periods. Because the vocalization peaks of eagle owls at sunset and sunrise could be more influenced by twilight [16], [22] than by lunar phase, for our analysis we excluded crepuscular call displays (i.e. the first hour after sunset and the first hour before sunrise). Differences among coefficients of dominance were analyzed using Friedman's Two-Way Analysis of Variance, because this index was not normally distributed and repeated measures were made on each owl (SPSS 15.0). Means are given ±SD, tests were two-tailed, and statistical significance was set at P<0.05.

## Results

**Figure 2:**
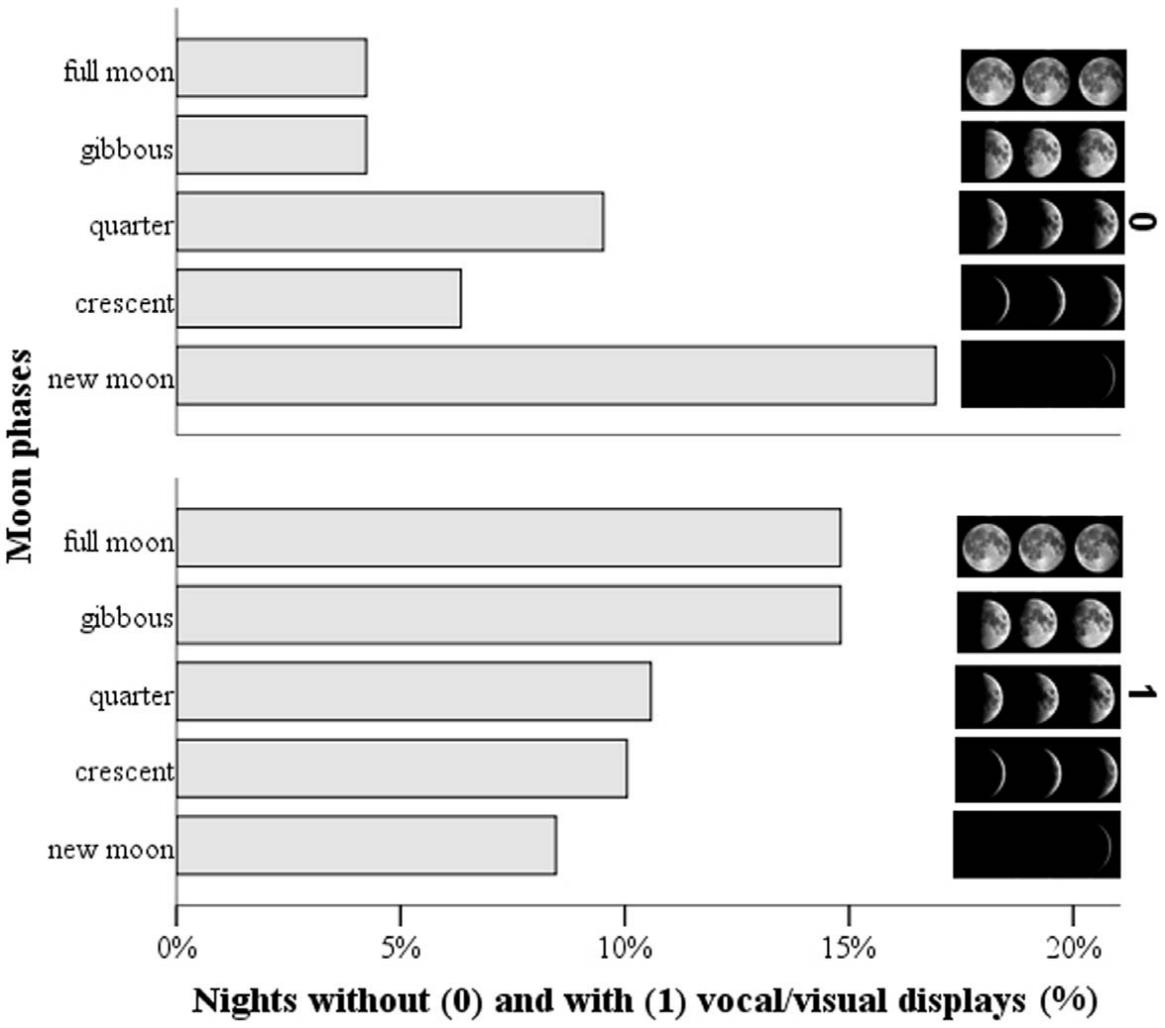
Call displays of eagle owls are strongly related to the moon phase: silent nights were more frequent among darker nights (e.g. new moon) compared to brighter nights (gibbous phase and full moon).

We found that the call displays of eagle owls are strongly related to the moon phase (F_4,182_ = 2.56, P = 0.04), in that silent nights were more frequent among darker nights compared to brighter nights (Fig. 2). This pattern was also influenced by sex (F_1,182_ = 6.38, P = 0.01): 80% of the vocal displays that we recorded came from males. The numbers of call bout series were explained by the moon phase (F_4,179_ = 27.09, P = 0.0001), sex (F_1,179_ = 41.56, P = 0.0001) and period (F_3,179_ = 34.97, P = 0.0001). The lowest numbers of bout series (range during dark nights = 1–4 series of call bouts per night) were only recorded during nights with no moon, while higher numbers of series (range during moonlit nights = 14–23 series of call bouts per night) were only recorded during the gibbous phase or at full moon. The additional effect of the breeding period and sex on the length of call displays is because they were mainly performed during the pre-laying period by males [22]. It is also worth noting that the frequency of crepuscular calls (sunset/sunrise displays strictly depend on ambient light [16]) were not related to the frequency of nocturnal vocal displays (χ^2^_1_ = 1.61, P = 0.20). In other words, eagle owls that called at sunset/sunrise did not continue to call (or called infrequently) in the absence of moonlight.

Finally, call posts were higher at moonlight (elevation above surroundings = 16.4±14.3 m) than at no-moon (elevation above surroundings = 11.0±14.5 m; χ^2^_2_ = 4.10, P = 0.0001). Under lunar brightness, higher positions would increase the conspicuousness of the white throat feathers that appear during the vocal displays of calling owls (Figure S1).

## Discussion

For species that signal using white patches, twilight represents among the best light conditions for visual communication with conspecifics [16]. Light levels of moonlight are similar to the light levels at dawn and dusk [18], when eagle owls perform the most vocalizations [16]. This could suggest that nocturnal birds simply take advantage of any source of natural light to increase the effectiveness of their visual communication.

This is not the first time that animal behaviors have been correlated to the amount of lunar brightness. It is important to highlight that, if moonlight affects communication, the specific effects maybe entirely dependent on the ecology of the species concerned. That is, birds that respond to moonlight conditions may show an opposite pattern to that shown by eagle owls. For example: (a) call frequencies of nocturnal seabirds have shown to be very low in moonlight and quickly increase when the moon was hidden by clouds [12]. The latter observation may indicate a direct relationship between predation pressure and light levels; and (b) although there is no detailed information about call display patterns for most owls, the Mexican spotted owl *Strix occidentalis lucida* called more during the last quarter and new moon phases [24]. While this is contrary to our present findings, it should be noted that Mexican spotted owls do not display white plumage while calling. Moreover, it would not be advantageous for them to call more at moonlight because moonlight calling could increase the risk of predation of this small owl by the bigger great horned owl *Bubo virginianus*.

To conclude, our results represent an additional example of how the moon can affect some animal behavior patterns. However, we have to acknowledge that the mechanism underlying the relationship between animal signaling and moonlight is not yet well understood.

## Supporting Information

Figure S1 The remarkable differences in conspicuousness of the white feathers of eagle owl throat badges on a moonlight night (A) and a dark night (B). During full moon periods, this visual signal is more visible and contrasts best with the background.
(0.32 MB PDF)
