# Supplementary material for: Moonlight Makes Owls More Chatty
Source: PLoS One. 2010 Jan 20;5(1):e8696. doi: 10.1371/journal.pone.0008696 (PMC2808345; doi:10.1371/journal.pone.0008696)

Supporting Information 1 (Figure S1)

The remarkable differences in conspicuousness of the white feathers of eagle owl throat badges on a moonlight night (A) and a dark night (B). During full moon periods, this visual signal is more visible and contrasts best with the background.

A

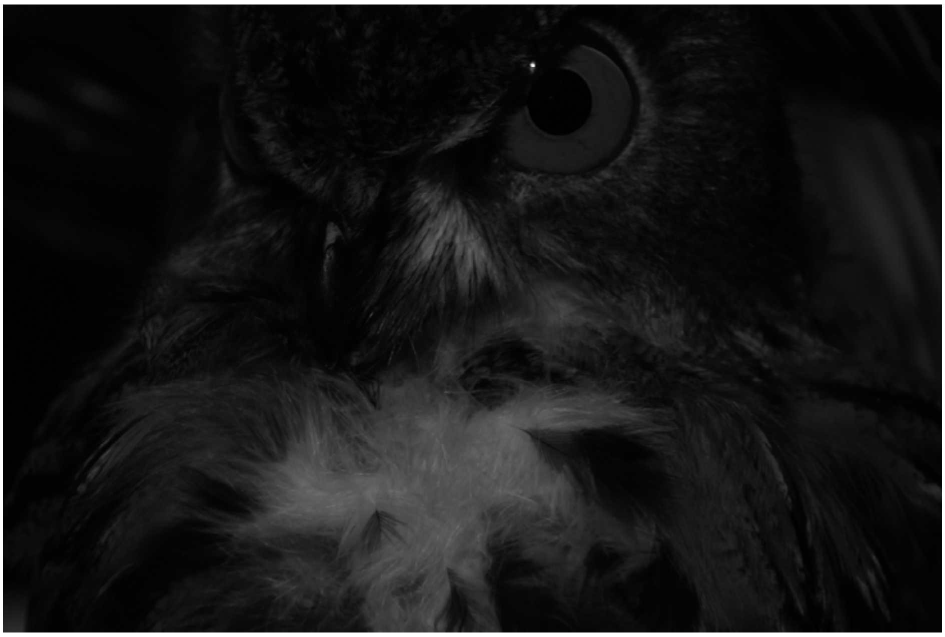

B

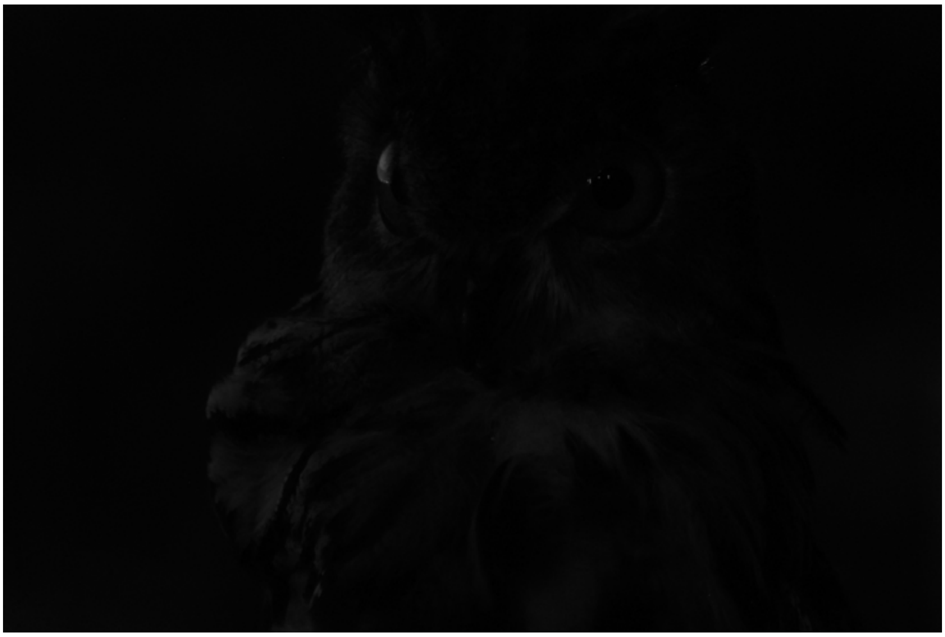

Supplement: Figure S1 — The remarkable differences in conspicuousness of the white feathers of eagle owl throat badges on a moonlight night (A) and a dark night (B). During full moon periods, this visual signal is more visible and contrasts best with the background. (0.32 MB PDF) [file pone.0008696.s001.pdf]
